# Uncommon presentation of primary hyperaldosteronism with severe hypomagnesemia: a Gitelman syndrome mimic

**DOI:** 10.1080/0886022X.2019.1662439

**Published:** 2019-09-09

**Authors:** Phatharaporn Kiatpanabhikul, Wasakorn Bunyayothin

**Affiliations:** a Department of Internal Medicine, Charoenkrung Pracharak Hospital, Bangkok Metropolitan Administration, Bangkok, Thailand;; b Department of Pathology, Charoenkrung Pracharak Hospital, Bangkok Metropolitan Administration, Bangkok, Thailand

**Keywords:** Hypomagnesemia, primary hyperaldosteronism, Gitelman syndrome, hypokalemia, hypertension

## Abstract

Primary hyperaldosteronism (PA) usually presents with moderate to severe hypertension with or without hypokalemia in adults. However, PA is not commonly associated with severe hypomagnesemia. By contrast, Gitelman syndrome usually presents with clinical manifestations of hypokalemia and hypocalcemia due to hypomagnesemia. Here, we present the case of a 44-year-old woman who first presented with peripheral paresthesia. Her laboratory tests revealed severe hypokalemia, metabolic alkalosis, severe hypomagnesemia, hypocalcemia and secondary hyperparathyroidism. The patient took high dose KCL tablets and Mg tablets to maintain normal values. She took only low-dose hydralazine to maintain normal blood pressure. Further investigations revealed PA with a left adrenal tumor. After left adrenalectomy, she remained in a normotensive, normokalemic and normomagnesemic state without any medical supplements. Thus, PA should be considered in patients with severe hypomagnesemia without moderate to severe hypertension.

## Introduction

Primary hyperaldosteronism (PA) usually presents with moderate to severe hypertension with or without hypokalemia in adults. However, PA is not commonly associated with severe hypomagnesemia [1]. By contrast, Gitelman syndrome (GS) usually presents with clinical manifestations of normotension, hypokalemia and hypocalcemia due to hypomagnesemia [2]. Here, we present a patient who was diagnosed with very severe hypomagnesemia and hypokalemic paresthesia with mild hypertension attributed to PA mimicking GS. We also discuss the differences in the clinical manifestations and pathogenesis between these two diseases.

## Case

A 44-year-old woman first presented with peripheral paresthesia after experiencing diarrhea twice in one day. She denied having nausea, vomiting, muscle cramping or weakness or periodic paralysis. The patient did not report the use of any drugs, including diuretics, licorice, herbal supplements and steroids. She had no family history of muscle weakness, early onset hypertension or cerebrovascular incidents at a young age. She was diagnosed with mild pre-eclampsia (BP 150/100 mmHg) during pregnancy at thirty years old. Three years later, she was diagnosed with hypertension (BP 156/112 mmHg) and took only low-dose combinations of two antihypertensive drugs (amlodipine 10 mg per day and losartan 50 mg per day) to control her blood pressure. Her electrolyte and magnesium (Mg) levels had never been tested. During her current presentation, her blood pressure was 125/72 mmHg. Physical examination revealed good consciousness, afebrility and no proximal muscle weakness in all extremities. She had Trousseau’s sign but no Chvostek’s sign. Her fundoscopic examination revealed no hypertensive retinopathy.

Her laboratory tests revealed severe hypokalemia (serum potassium 1.6 mmol/L; normal range 3.5–5.1), metabolic alkalosis (serum bicarbonate 36 mmol/L; normal range 18–24), severe hypomagnesemia (serum Mg 0.9 mg/dL; normal range 1.6–2.6), hypocalcemia (total calcium 7.1 mg/dL; normal range 8.6–10.2) with a response consistent with hyperparathyroidism (intact parathyroid hormone (PTH) 98.9 pg/ml; normal range 15–68.3). Electrocardiography showed a prolonged QT interval (NSR 84/min with QTc 565 msec). Normal or negative tests included sodium chloride, blood urea nitrogen (BUN), creatinine, liver function and thyroid function tests. After intravenous administration of 10% calcium gluconate, 50% Mg phosphate and oral potassium chloride (KCL) improved her peripheral paresthesia and Trousseau’s sign but could not maintain normal potassium and Mg levels. The patient took a KCL tablet (8000 mg per day) and Mg tablet (300 mg per day) to maintain normal values. She took hydralazine (50 mg per day) to maintain normal blood pressure. Further investigation showed renal potassium and Mg loss (urine potassium creatinine ratio, UKCR 165 mmol/gCr; normal range <20 and 24-h urine Mg 3.5 mmol/day; normal range <1) with hypocalciuria (24-h urine Ca 2.1 mg/day; normal range 20–275). Renal potassium wasting, hypokalemia, metabolic alkalosis, and hypomagnesemia with hypocalciuria prompted us to suspect GS^2^. However, because this syndrome mostly appears in younger patients with normotension, PA was considered and confirmed by plasma aldosterone 69.7 ng/dL (normal range <15), direct renin 0.9 uIU/L (normal range 6.5–45) and plasma aldosterone to direct renin ratio (ARR) 77.4 (Table 1).

**Table 1.** Patient blood pressure, important laboratory values and treatments during management.

Parameter (normal range)	At first visit	During work up		Postop 1 day	Postop 2 weeks	Postop 6 weeks	Postop 3 months	Postop 6 months	Postop 18 months	Treatment and notes
BP, mmHg	125/72	148/92	Laparoscopic left adrenalectomy	139/89	114/75	127/84	127/80	117/86	122/88	Hydralazine 50 mg/day then off after surgery
BUN (6–20 mg/dL)	7	11		11	19	22	18	18	14	–
Creatinine (0.5–0.9 mg/dL)	0.71	0.82		0.62	0.92	1.27	1.23	1.10	1.06	–
Potassium (3.5–5.1 mmol/L)	1.6	3.6		4.2	3.9	4.6	4.1	4.5	3.9	Potassium Chloride tab 8000 mg/day then off after surgery
Bicarbonate (18–24 mmol/L)	36	29		22	22	21	24	22	20	–
Magnesium (1.6–2.6 mg/dL)	0.9	1.6		1.6	1.8	2.0	2.0	2.0	2.0	Magnesium tab 300 mg/day then off after surgery
Total calcium (8.6–10.2 mg/dL)	7.1	9.1		–	–	–	–	–	9.8	–
Albumin (4.0–5.0 g/dL)	3.9	4.4		–	–	–	–	–	4.5	
Phosphate (2.5–4.5 mg/dL)	3.3	3.3		–	–	–	–	–	3.5	
PTH (15–68.3 pg/ml)	98.9	–		–	–	–	–	–	61.9	–
Aldosterone (A) (<15 ng/dL)	–	69.7		19.5	–	–	10.8	–	–	–
Renin –direct (R) (6.5–45 uIU/L)	–	0.9		–	–	–	7.2	–	–	–
ARR (<2.4)	–	77.4		–	–	–	1.5	–	–	–
Urine Albumin-to-Creatinine Ratio (<30 mg/gCr)	43.7	–		–	–	–	–	–	4	–

**Figure 1.**
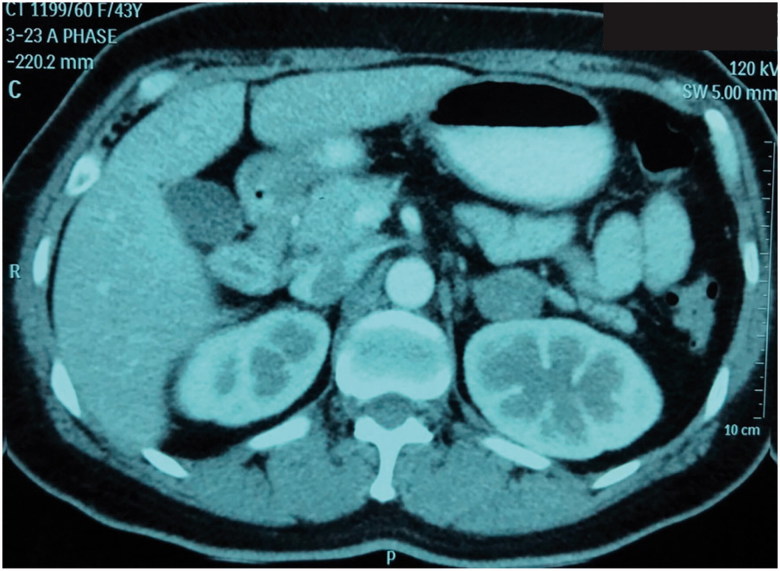
This figure shows a 2.0 × 2.7 cm well-defined enhancing hypodense nodule in the lateral limb of the left adrenal gland. The measured density on precontrast images is approximately 6–12 HU. The calculated washout is approximately 82%, likely left adrenal adenoma (arterial phase CT). The right adrenal gland appeared normal.

**Figure 2.**
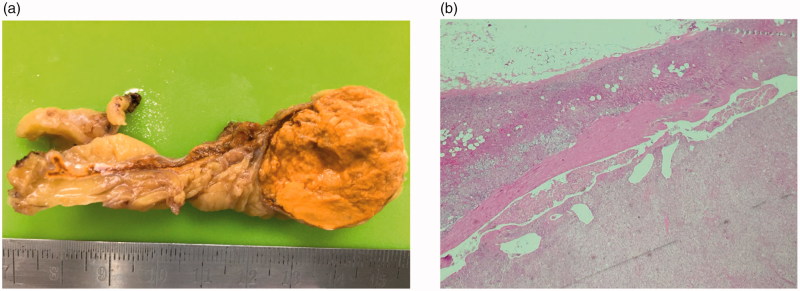
(a) Left adrenal gland measured 6 × 2.5 × 2 cm and weighed 9 gm. The tumor measured 3 × 2×1.5 cm and was located at the tail of the adrenal gland. The tumor was golden yellow and well-demarcated. (b) Microscopically, a partial fibrous encapsulated mass consisting of polygonal cells with an organoid pattern. No atypical mitosis was found. Subcapsular sinusoidal invasion was encountered. Neither lymph-vascular invasion nor capsular invasion was demonstrated.

A multiphase computed tomography (CT) adrenal protocol showed a 2.0 × 2.7-cm, well-defined enhancing hypodense nodule in the lateral limb of the left adrenal gland, likely adrenal adenoma (Figure 1). Her diagnosis was PA with left aldosterone-producing adenoma (APA). After laparoscopic left adrenalectomy, the pathology confirmed a benign adrenal cortical adenoma with complete resection (Figure 2). Although her postoperative plasma aldosterone level was not lower than 5–19.5 ng/dl, the patient remained in a normotensive, normokalemic and normomagnesemic state without any medications for at least 18 months of follow up. The restoration of plasma aldosterone, direct renin, Mg level, calcium level and PTH level is shown in Table 1.

## Discussion

GS is an autosomal recessive disorder characterized by renal potassium wasting, hypokalemia, metabolic alkalosis, hypomagnesemia with hypocalciuria and normotension in children and young adults. These patients often present with asymptomatic or muscular cramps or nonspecific paresthesia and weakness [2]. The prevalence of GS was 1:40 000 which was caused by mutation of genes encoding sodium chloride cotranspoters and Mg channels in the distal convoluted tubule [3]. The major pathophysiology of GS comprises inhibited expression of the transient receptor potential melastatin 6 (TRPM6) channel, which reabsorbs urinary Mg; this reduced expression contributes hypomagnesemia and exacerbated salt loss to cause hyperreninemic hyperaldosteronism leading to subsequent hypokalemic metabolic alkalosis [3,4].

PA involves an inappropriately high of aldosterone production for sodium status that is relatively independent of the major regulators of secretion (angiotension II and plasma potassium) and cannot be suppress by sodium loading. These patients commonly present with moderate to severe hypertension with normokalemia to hypokalemia; most likely, more severe cases in adults are caused by hyporeninemic hyperaldosteronism [1]. In our case, an uncommon clinical manifestation of PA was normotension. A previous cohort study showed a spectrum of PA; specifically, more renin suppression with higher aldosterone concentrations was associated with lower serum potassium, higher urinary excretion of potassium and independently associated with an increased risk for incident hypertension [5]. There are other syndromes involving mineralocorticoid excess with low renin concentrations such as Cushing’s syndrome, glucocorticoid/cortisol resistance, apparent mineralocorticoid excess syndrome, excess licorice or carbenoxolone, congenital adrenal hyperplasia (11 beta- and 17 alpha-hydroxylase deficiencies), 11-deoxycorticosterone (DOC), 18-hydroxy-DOC excess, Geller syndrome, Gordon’s syndrome and Liddle’s syndrome which have more specific clinical findings and clues to identify patients with hypertension [6].

Acute hypomagnesemia and hypokalemic peripheral paresthesia are not common clinical manifestations of PA. Our patient had the same presentation and blood test abnormalities as those observed in GS. The only clinical manifestation that differentiated this disease was hypertension treatment for ten years. When plasma aldosterone and renin were examined, the tests revealed hyporeninemic hyperaldosteronism, by contrast, patients with GS usually present with high levels of plasma renin. Thus, our patient was diagnosed with PA. Mild hypomagnesemia due to urinary Mg wasting may also occur in patients with persistent mineralocorticoid excess. However, in PA, hypomagnesemia is not a common manifestation and is not usually as severe as that observed in our patient. There are other secondary causes of hypomagnesemia, such as chronic diarrhea, diabetic ketoacidosis, alcoholism, diuretics and nephrotoxins (amphoteracin B, aminoglycosides), but none of these contributing conditions or medications were applicable to our patient. Few previous studies have shown an unusual presentation of PA, similar to that of our patient. In an earlier published case, a 50-year-old woman from Turkey in 2009 [7] presented with sudden onset progressive paralysis involving four extremities, and her blood chemistries also revealed severe hypokalemia, severe hypomagnesemia and hypocalcemia with secondary hyperparathyroidism. The difference between this patient and our patient was normotension. However, her age was older than usual ages of patients with GS, and PA was taken into consideration. In a recently published case, a 29-year-old woman from Malaysia in 2017 [8], presented with peripheral numbness. She also had hypokalemia, hypomagnesemia and hypocalcemia but did not present severe or secondary hyperparathyroidism as observed in our patient. Although this patient was younger, her hypertension appeared one year after her initial presentation, prompting the investigation of PA. In contrast to our patient, she had hypercalciuria, which was the proposed mechanism related to expansion of the extravascular space, resulting in decreased proximal tubular reabsorption and thereby increased distal delivery of Na^+^, Mg^2+^, Ca^2+^ with mineralocorticoids promoting distal tubular Na + reabsorption without impairing Mg and calcium excretion [9]. However, our patient had hypocalciuria, similar to GS. How this condition occurs is incompletely understood. The ascending limb of Henle’s loop is the primary site of tubular Mg reabsorption, and inhibition of sodium transport in this segment during aldosterone escape may be associated with a parallel decline in Mg reabsorption [10]. In a previous report, the measured intracellular concentration of ionized Mg was significantly lower in 16 patients with PA than in normotensive control subjects. These data can support and explain how aldosterone affects the cellular homeostasis of Mg: probably through modification of Na^+^-Mg^2+^ antiporter activity [11].

## Conclusion

PA should be considered in patients with severe hypomagnesemia combined with hypokalemic metabolic alkalosis without moderate to severe hypertension. Persistent mineralocorticoid excess may contribute to urinary Mg wasting.
